# Advances in Reperfusion Therapy and Cytoprotection for Acute Ischemic Stroke

**DOI:** 10.3390/jcdd13060229

**Published:** 2026-05-27

**Authors:** Zihan Li, Chunjuan Wang

**Affiliations:** 1 Department of Neurology, Beijing Tiantan Hospital, Capital Medical University, Beijing 100069, China; lizihan123@mail.ccmu.edu.cn; 2 China National Clinical Research Center for Neurological Diseases, Beijing Tiantan Hospital, Capital Medical University, Beijing 100069, China

**Keywords:** acute ischemic stroke, thrombolysis, thrombectomy, reperfusion therapy, cytoprotective agents, review

## Abstract

Stroke is one of the leading causes of disability and mortality worldwide, and approximately 87% of cases are acute ischemic stroke (AIS). For patients with AIS, rapid administration of reperfusion therapy within the therapeutic time window remains the most effective treatment strategy. Over the past decade, numerous high-quality clinical trials have driven rapid advances in treatment strategies. Meanwhile, increasing attention has been directed toward cytoprotective therapies aimed at mitigating ischemic and reperfusion-related brain injury, which may act synergistically with reperfusion strategies. Although many related clinical trials have failed to demonstrate clear clinical benefit, they have provided valuable insights for the development of future cytoprotective agents. This review focuses on recent advances and remaining challenges in reperfusion therapy and cytoprotection for AIS.

## 1. Introduction

Stroke remains a leading cause of disability and mortality worldwide, with acute ischemic stroke (AIS) accounting for approximately 87% of all stroke cases [1]. AIS results from an abrupt reduction in cerebral blood flow, typically due to thrombosis or embolism, leading to ischemia, hypoxia, and ultimately infarction of affected brain tissue. Its etiology is multifactorial, with common causes including atherosclerotic plaque rupture, cardioembolism, small-vessel disease, and various vascular or hemodynamic abnormalities. Regardless of the underlying mechanism, cerebral ischemia rapidly causes neuronal injury, and delayed reperfusion is strongly associated with permanent neurological deficits and poor functional outcomes [2]. Over the past three decades, AIS management has focused primarily on reperfusion strategies targeting the occluding thrombus [3]. Currently, intravenous thrombolysis (IVT) and endovascular thrombectomy (EVT) are the primary methods for achieving vascular recanalization and restoring cerebral blood flow. These approaches have been validated in large-scale randomized controlled trials, resulting in substantial improvements in clinical outcomes and widespread adoption in routine practice [4]. In parallel, cytoprotective approaches that aim to attenuate ischemic and reperfusion-induced injuries are also being actively investigated as potential adjuncts to reperfusion therapy.

**Figure 1:**
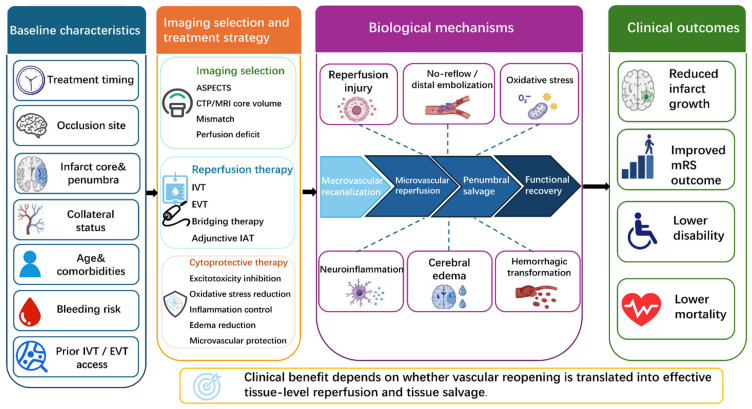
Integrated reperfusion–cytoprotection framework for tissue-level salvage in acute ischemic stroke.

In this review, we summarize recent advances in reperfusion therapy and cytoprotective strategies for AIS, with a focus on pivotal randomized controlled trials published in recent years. Beyond reviewing the available evidence, we examine why similar therapeutic approaches have produced divergent results across trials and how modern AIS treatment is moving beyond vessel reopening alone toward more precise tissue salvage. We further discuss how reperfusion strategies, cytoprotection, imaging-based selection, and patient heterogeneity can be integrated to inform more tissue-oriented AIS treatment (Figure 1).

Baseline patient heterogeneity and imaging selection influence treatment eligibility, therapeutic strategy, and the likelihood of tissue salvage. Reperfusion therapies, including IVT, EVT, bridging therapy, and adjunctive IAT, aim to restore macrovascular blood flow, whereas cytoprotective strategies may attenuate excitotoxicity, oxidative stress, inflammation, edema, and microvascular injury. The clinical benefit of treatment ultimately depends on whether vascular reopening and reperfusion quality can be translated into effective microvascular reperfusion, penumbral salvage, functional recovery, and improved clinical outcomes. Dashed arrows indicate biological factors that may modulate the transition from macrovascular recanalization to microvascular reperfusion, penumbral salvage, and functional recovery.

## 2. Intravenous Thrombolysis for Acute Ischemic Stroke

Since its approval in 1996, IVT has become a critical treatment for AIS. IVT (with alteplase) administered within 4.5 h of symptom onset remains the most widely recommended pharmacological therapy and is endorsed by major international guidelines [5]. Nevertheless, limitations such as supply shortages [6], the need for weight-based dosing and prolonged infusion time have prompted numerous clinical trials to investigate novel thrombolytic agents and explore the extension of the therapeutic time window.

### 2.1. New Thrombolytic Agents

Tenecteplase is a genetically modified variant of human tissue plasminogen activator (tPA), differing from alteplase by three amino acid substitutions: T103N, N117Q, and KHRR296–299AAAA [7]. Because it is resistant to inactivation by plasminogen activator inhibitor-1 and has a longer half-life [8], it can be administered as a single intravenous bolus, offering practical advantages over alteplase.

Early studies provided important insights into optimal dosing. In the NOR-TEST 1 trial, a dose of 0.4 mg/kg tenecteplase was found to be safe but not superior to alteplase in patients with predominantly mild strokes [9]. However, the phase 3 NOR-TEST 2 trial was terminated early when this dose (0.4 mg/kg) resulted in higher rates of symptomatic intracranial hemorrhage (sICH) and worse functional outcomes compared to 0.9 mg/kg alteplase, establishing this dose as unfavorable [10]. The TEMPO-1 study further demonstrated improved recanalization with tenecteplase at 0.25 mg/kg compared with 0.1 mg/kg, leading subsequent trials to adopt the 0.25 mg/kg regimen [11].

Over the past five years, several large phase 3 randomized controlled trials have directly compared the efficacy and safety of tenecteplase (0.25 mg/kg) versus alteplase (0.9 mg/kg) (Table 1) [12,13,14,15,16], consistently demonstrating the non-inferiority of tenecteplase relative to alteplase. Most clinical trial subgroup analyses have shown that the thrombolytic efficacy and safety of tenecteplase, compared with alteplase, are generally consistent across major clinical subgroups such as age and sex, with no significant heterogeneity observed between subgroups. Notably, the ORIGINAL trial suggested a greater trend toward benefit with tenecteplase in patients older than 80 years [16], whereas the TASTE trial indicated that its efficacy signal might be more pronounced in patients with distal anterior circulation occlusions, but not in those with large-vessel occlusion (LVO) [15]. The reasons for these findings remain unclear but may relate to the simpler single-bolus administration of tenecteplase, which could facilitate earlier reperfusion, as well as differences in study populations and imaging selection strategies across trials. A 2024 systematic review and meta-analysis (*n* = 7545), which pooled data from 11 randomized controlled trials, provided further support for tenecteplase as an alternative to alteplase within the 4.5 h time window. Tenecteplase was associated with a higher likelihood of excellent functional outcome at 90 days (mRS 0–1: risk ratio [RR] 1.05, 95% CI 1.01–1.10), without significant differences in rates of sICH or mortality [17]. Collectively, these findings establish tenecteplase at a dose of 0.25 mg/kg as an effective and safe thrombolytic agent across diverse patient populations and provide a strong evidentiary basis for future guideline development.

Besides alteplase and tenecteplase, additional thrombolytic agents continue to be studied. Reteplase, which is used in cardiovascular disease for the treatment of acute myocardial infarction, is a recombinant plasminogen activator that is characterized by a double-bolus approach (the boluses are separated by 30 min) with a fixed dose regimen [18,19]. A randomized controlled non-inferiority trial (RAISE) compared two 18-mg boluses of reteplase with standard alteplase in 1412 patients with AIS eligible for IVT within 4.5 h of symptom onset [20]. At 90 days, a higher proportion of patients in the reteplase group achieved good functional outcome (mRS 0–1: 79.5% vs. 70.4%, RR 1.13, 95% CI 1.05–1.21), meeting the prespecified non-inferiority criterion, but the incidence of any intracranial hemorrhage was higher in the reteplase group than in the alteplase group (7.7% vs. 4.9%, RR 1.59, 95% CI 1.00–2.51). These findings suggest that, although reteplase has demonstrated potential as a thrombolytic agent, its associated bleeding risk should not be underestimated and may partially offset its therapeutic benefit. Therefore, further studies are warranted to better define its safety profile and overall risk–benefit balance. In clinical practice, particular caution is also needed regarding its potential for increased hemorrhagic risk.

Like reteplase, recombinant human prourokinase (rhPro-UK) has emerged as a promising thrombolytic agent. As a fibrin-specific plasminogen activator, prourokinase exhibits thrombolytic efficacy comparable to alteplase, with a potentially lower risk of bleeding [21,22]. The exploratory phase IIa PROST trial (*n* = 119) compared the efficacy and safety of low-dose (35 mg) and high-dose (50 mg) rhPro-UK with alteplase in Chinese patients with AIS within 4.5 h of onset. The study ultimately found that both low- and high-dose prourokinase showed no significant differences in efficacy or safety compared to alteplase (*p* = 0.92) [21]. The phase 3 non-inferiority PROST-2 study compared rhPro-UK (35 mg) with alteplase (0.9 mg/kg) in 1552 patients with AIS within 4.5 h of symptom onset [22]. At 90 days, 72.0% of patients in the rhPro-UK group achieved mRS 0–1 compared with 68.7% in the alteplase group (RR 1.04, 95% CI 0.98–1.10), meeting the prespecified non-inferiority criterion. Moreover, there was a lower occurrence of sICH within 36 h (0.3% vs. 1.3%, risk difference [RD] 1.0%, 95% CI −2.1 to 0.1) and mortality within 7 days (0.6% vs. 1.7%, RD 1.0%, 95% CI −2.3 to 0.1) in the rhPro-UK group than in the alteplase group. Together, these studies support rhPro-UK as an effective thrombolytic option with a favorable safety profile, accelerating its clinical adoption.

Against the backdrop of the continued global evolution of IVT strategies for AIS and the increasing diversification of available thrombolytic agents, the Center for Drug Evaluation (CDE) of China’s National Medical Products Administration has successively approved rhTNK-tPA (tenecteplase biocopy manufactured in China and used in TRACE-2), tenecteplase, reteplase, and rhPro-UK for IVT in AIS, reflecting a transition of IVT therapy from reliance on a single dominant agent to a “multi-drug era”. This development reflects the emergence of a broader new treatment paradigm in IVT for AIS.

### 2.2. Intravenous Thrombolysis with an Extended Time Window

Since the ECASS-3 study in 2008 extended the IVT time window for AIS to 4.5 h [23], this threshold has remained the standard for many years. The EXTEND trial later demonstrated that, with multimodal imaging selection, selected patients could benefit from thrombolysis up to 9 h after symptom onset, although routine clinical practice has largely continued to adhere to the 4.5 h window [24]. Recent randomized trials using advanced imaging techniques have further explored the feasibility of extending treatment to 24 h in carefully selected patients.

In 2024, the phase 3 TIMELESS trial evaluated the efficacy of tenecteplase administered between 4.5 and 24 h after stroke onset in patients with LVO [25]. Although a trend toward clinical benefit was observed, the results did not reach statistical significance, possibly because more than 70% of participants also underwent EVT, which may have attenuated the detectable effect of thrombolysis. The ETERNAL-LVO trial also did not demonstrate a significant benefit of tenecteplase on 90-day functional outcomes in patients with anterior-circulation LVO presenting within 24 h and selected based on perfusion imaging [26]. However, the trial was terminated early because of drug shortages and the rapidly evolving evidence landscape for tenecteplase in the early time window, rendering it underpowered and precluding definitive conclusions. Notably, a more favorable signal was observed in transferred patients, suggesting that tenecteplase may be of greater relevance in settings where access to EVT is delayed. In contrast, the TRACE-3 trial, in which most patients did not receive EVT, demonstrated a higher proportion of patients achieving the absence of disability (mRS 0–1) at 90 days in the tenecteplase group (33.3% vs. 24.2%, relative rate 1.37, 95% CI 1.04–1.81, *p* = 0.03), despite an increased risk of sICH [27]. Collectively, these studies suggest that the role of tenecteplase beyond 4.5 h may depend largely on thrombectomy access, with the clearest signal of benefit observed in patients who are not candidates for EVT or in whom EVT is delayed.

Two additional randomized trials further supported extended-window thrombolysis. The EXPECTS trial, which focused on patients with mild posterior circulation ischemic stroke (median NIHSS score, 3) who were eligible for IVT between 4.5 and 24 h after symptom onset, indicated that alteplase thrombolysis significantly increased the rate of functional independence at 90 days versus standard medical care (mRS 0–2: 89.6% vs. 72.6%, adjusted RR 1.16, 95% CI 1.03–1.30, *p* = 0.01), without a significant increase in 90-day mortality (5.2% vs. 8.5%, unadjusted RR, 0.61, 95% CI 0.23–1.62), although the risk of sICH was higher [28]. Similarly, the HOPE trial enrolled patients with more severe strokes (median NIHSS score, 10) and imaging-confirmed ischemic penumbra, and likewise demonstrated a benefit of alteplase [29]. Notably, the HOPE study used widely available commercial post-processing imaging software, enhancing the generalizability of its findings. Taken together, the EXPECTS and HOPE trials provide complementary evidence that alteplase may remain beneficial beyond 4.5 h in carefully selected patients, ranging from mild posterior circulation stroke to more severe ischemic stroke with imaging-defined penumbra.

Recently completed trials continue to refine IVT therapy strategies and optimize patient selection. The OPTION trial showed that, in patients with AIS presenting 4.5–24 h after onset, without LVO but with salvageable tissue on perfusion imaging, IVT (tenecteplase) significantly increased the proportion achieving an excellent 90-day outcome (mRS 0–1) versus standard medical treatment (43.6% vs. 34.2%, RR 1.28, 95% CI 1.04–1.57, *p* = 0.02), although with a higher risk of sICH [30]. In addition, the TRACE-5 trial demonstrated that patients with basilar artery occlusion (BAO) presenting within 24 h can benefit from tenecteplase (38% vs. 29%, adjusted relative rate 1.50, 95% CI 1.09–2.08, *p* = 0.014), in a clinical context allowing EVT, without increasing sICH or 90-day mortality [31]. Together, these recent trials suggest that IVT beyond 4.5 h may be beneficial in carefully selected AIS patients with imaging evidence of salvageable ischemic tissue, regardless of LVO status. This strategy may be particularly important for patients with LVO who are not eligible for EVT or in whom EVT access is delayed, because IVT may offer an opportunity for tissue salvage when EVT cannot be performed promptly. These findings also support broadening the potential population eligible for extended-window IVT and provide new evidence for individualized reperfusion strategies across different occlusion subtypes.

## 3. Endovascular Thrombectomy

EVT has emerged as a pivotal therapeutic modality for AIS. Compared with IVT, EVT offers a wider treatment time window and achieves superior recanalization rates in patients with LVO. Currently, EVT is considered the standard treatment for AIS with LVO. In 2015, five landmark clinical trials (MR CLEAN, ESCAPE, REVASCAT, SWIFT PRIME, and EXTEND IA) demonstrated that EVT was superior to IVT alone in patients with proximal anterior circulation occlusion [32,33,34,35,36], thereby providing definitive evidence supporting EVT as a cornerstone therapy and fundamentally transformed the management paradigm for LVO-related stroke. Over the past five years, research in EVT has focused on extending the therapeutic time window, expanding eligibility criteria with respect to infarct core size, and optimizing treatment strategies involving bridging and direct thrombectomy.

### 3.1. Extended Time Window

Early randomized trials establishing the efficacy of mechanical thrombectomy predominantly enrolled patients within 6 h of the last known well [37]. Subsequent pivotal studies, including the DAWN and DEFUSE-3 trials, extended the therapeutic window to 24 h in selected patients, providing high-level evidence supporting EVT in the late time window (Table 2) [38,39]. Both trials consistently demonstrated that patients with anterior-circulation LVO could still derive substantial benefit from EVT beyond 6 h after stroke onset (for DAWN, adjusted difference 33%, 95% CI 21–44; for DEFUSE 3, odds ratio 2.77, *p* < 0.001). The DAWN trial showed that, under strict selection based on a mismatch between clinical deficit severity and infarct core volume, the treatment effect of EVT was generally consistent across prespecified subgroups, with no significant heterogeneity observed [39]. The DEFUSE 3 trial further showed that the benefit of EVT was not meaningfully attenuated even in a broader population than that enrolled in DAWN, suggesting that, among imaging-selected patients in the late time window, the therapeutic effect of EVT remains relatively robust [38].

While the evidence base for extended-window EVT in anterior circulation stroke is well established, substantial progress has also been made in posterior circulation stroke (Table 2). The ATTENTION trial (*n* = 340) demonstrated that EVT combined with best medical therapy was superior to medical therapy alone in patients with BAO treated within 12 h of onset. A favorable functional outcome (mRS 0–3 at 90 days) was achieved in 46% of patients in the EVT group compared with 23% in the control group (adjusted rate ratio 2.06, 95% CI 1.46–2.91, *p* < 0.001) [40]. Similarly, the BAOCHE trial (*n* = 217) enrolled patients with BAO presenting within 6–24 h and without large baseline infarction, confirming a significant functional benefit of EVT at 90 days (46% vs. 24%, adjusted RR 1.81, 95% CI 1.26–2.60, *p* < 0.001) [41]. Collectively, these trials support extending the EVT time window up to 24 h after onset for AIS involving both anterior and posterior circulation LVO. These advances have been incorporated into recent clinical guidelines, as reflected in the 2026 Guideline for the Early Management of Patients with Acute Ischemic Stroke, which explicitly recommends a treatment window of up to 24 h [5].

Pushing the time window further, the retrospective observational SELECT late study (*n* = 301) found that EVT beyond 24 h was associated with higher functional independence than medical management (38% vs. 10%, inverse probability treatment weighting adjusted odds ratio [IPTW aOR] 4.56, 95% CI 2.28–9.09, *p* < 0.001), despite higher odds of sICH (10.1% vs. 1.7%, IPTW aOR 10.65, 95% CI 2.19–51.69, *p* = 0.003) [42]. The study further suggested that patients with higher Alberta Stroke Program Early Computed Tomography Score (ASPECTS), smaller ischemic core volumes, and the presence of perfusion mismatch were more likely to benefit from EVT, whereas in patients presenting in the ultra-late time window with more advanced baseline ischemic changes, the risk of hemorrhage might progressively offset its potential benefit. This issue still requires confirmation in prospective studies and may also inform refinement of eligibility criteria in future randomized trials, for example, by applying more cautious selection to patients with very low ASPECTS or those presenting well beyond 24 h.

### 3.2. Benefits of Thrombectomy in Large-Core Infarction

Most randomized controlled trials of EVT have focused on patients with AIS caused by LVO with small infarct cores, whereas evidence in those with large core infarcts was limited. Large-core infarct is typically defined as anterior circulation LVO with ASPECTS of 3–5 or an infarct-core volume of 50–100 mL. In recent years, several high-quality phase 3 trials have addressed this previously understudied population. A series of randomized trials consistently demonstrated that EVT provides meaningful clinical benefit in patients with large ischemic cores compared with medical management alone (Table 2) [43,44,45,46,47].

A 2025 meta-analysis further strengthened the evidence that EVT confers functional benefit in patients with large-core infarction, with the most robust evidence seen in those treated within 6 h of onset, with ASPECTS 3–5, and with occlusion located in the intracranial internal carotid artery (ICA) or proximal M1 segment of the middle cerebral artery (MCA) [48]. Although extended-window trials, such as ANGEL-ASPECT and SELECT2, also showed that EVT improved functional outcomes compared with medical management alone, their findings on safety and mortality were not entirely consistent [44,49], suggesting that the balance between benefit and risk may be more complex in patients treated in later time windows. Notably, the TESLA trial failed to meet its primary endpoint, which may be partly explained by differences in patient selection, imaging criteria, and trial design rather than by an intrinsic lack of EVT efficacy [47]. Although TESLA enrolled patients within 24 h, nearly half were treated in the 12–24 h window, when more advanced edema, microvascular dysfunction, and irreversible tissue injury may have attenuated the benefit of reperfusion. In addition, TESLA used noncontrast computed tomography (NCCT)-based ASPECTS 2–5 selection, thereby including patients with very low ASPECTS and potentially more severe infarction than trials using ASPECTS 3–5 or additional core-volume/perfusion criteria. The numerically lower rate of successful reperfusion in TESLA may also have limited the translation of recanalization into clinical benefit. Finally, the use of utility-weighted mRS as the primary endpoint may have influenced the statistical interpretation of treatment benefit. Thus, TESLA suggests that the benefit of EVT in patients with large-core infarction is conditional, highlighting the need to better define the optimal treatment window, imaging selection strategy, reperfusion-quality targets, and potential role of bridging therapy in this population.

### 3.3. Bridging and Adjunctive Thrombolytic Therapy

Although both IVT and EVT are established treatments for AIS, the optimal sequencing of these therapies remains under active investigation. Multiple randomized trials published between 2018 and 2023 comparing bridging therapy (IVT followed by EVT) with EVT alone did not demonstrate clear superiority of the combined approach [50]. However, the 2025 BRIDGE-TNK trial (*n* = 278) suggested potential benefit in selected patients, showing that intravenous tenecteplase before EVT increased the likelihood of functional independence (mRS 0–2) at 90 days compared with EVT alone (52.9% vs. 44.1%, RR 1.20, 95% CI 1.01–1.43, *p* = 0.04) [51].

Increasing attention has also focused on adjunctive thrombolytic therapy, in which intra-arterial thrombolysis (IAT) is administered after successful EVT (Table 3). The CHOICE trial first explored this strategy and, despite early termination, suggested improved functional outcomes with adjunctive thrombolytic therapy after successful reperfusion [52]. Subsequent randomized trials, however, have not shown a consistent benefit of adjunctive IAT. In 2024, two randomized controlled trials (POST-UK, *n* = 532; POST-TNK, *n* = 539) showed that, after successful reperfusion with EVT, additional IAT did not provide a clear incremental benefit compared with EVT alone (POST-UK: RR 1.13, 95% CI 0.94–1.36, *p* = 0.19; POST-TNK: RR 1.15, 95% CI 0.97–1.63, *p* = 0.11). This may be partly because the enrolled patients had already achieved near-complete or complete reperfusion after EVT, leaving limited room for further benefit from adjunctive thrombolysis [53,54]. In contrast, the ANGEL-TNK trial (*n* = 256) demonstrated that adjunctive thrombolytic therapy was associated with a greater likelihood of excellent neurological outcome at 90 days (40.5% vs. 26.4%, relative risk 1.44, 95% CI 1.06–1.95, *p* = 0.02), without increasing the risk of sICH or mortality [55]. Similarly, the PEARL trial suggested that IAT with alteplase after successful EVT reperfusion might further improve 90-day functional outcomes (44.8% vs. 30.2%, adjusted RR 1.45, 95% CI 1.08–1.96, *p* = 0.01) [56]. One possible explanation is that trials such as PEARL and ANGEL-TNK used relatively broad definitions of successful reperfusion, such as expanded Thrombolysis in Cerebral Infarction (eTICI) ≥ 2b or eTICI ≥ 2b50, thereby including patients who may still have residual downstream perfusion deficits, potentially related to distal embolization or incomplete tissue-level reperfusion despite angiographic success. This may have left greater room for adjunctive IAT to improve microvascular reperfusion and salvage viable tissue within regions initially classified as infarcted on imaging. However, the phase 1b/2a DATE trial (*n* = 48/157) evaluated IAT with tenecteplase after successful EVT and found that doses of 0.0313 mg/kg or 0.0625 mg/kg were adequately safe but did not demonstrate significant functional benefit, possibly owing to limited sample size [57]. Overall, the available data provide a more coherent efficacy signal for alteplase-based adjunctive IAT than for intra-arterial tenecteplase, although direct comparisons are lacking. The heterogeneous findings for tenecteplase warrant further large-scale clinical studies to clarify the efficacy and safety of different doses of tenecteplase as an adjunct to EVT.

In posterior circulation stroke, the ATTENTION-IA trial (*n* = 208) found that adjunctive thrombolytic therapy did not significantly improve the rate of functional independence compared with EVT alone (34.6% vs. 26.0%, adjusted RR 1.36, 95% CI 0.92–2.02, *p* = 0.12) [58]. This neutral result may partly reflect the distinct clinical behavior of posterior circulation stroke, in which small brainstem or perforator-territory lesions can cause severe disability, making angiographic reperfusion less directly translatable into excellent functional recovery. The numerically higher rate of sICH further suggests a narrower therapeutic margin for adjunctive thrombolysis in this setting.

Nevertheless, a pooled analysis of six randomized trials showed that adjunctive IAT after successful EVT was associated with improved odds of excellent functional outcome (mRS 0–1) at 90 days (odds ratio 1.47, 95% CI 1.21–1.80, *p* < 0.001), without a significant increase in sICH or 90-day mortality [59].

Collectively, current evidence suggests that adjunctive IAT after successful EVT remains a potentially promising but unsettled strategy. Its benefit appears more evident in anterior-circulation stroke, alteplase-based regimens, and patients with residual downstream reperfusion deficits, whereas uncertainty remains in posterior-circulation stroke, tenecteplase-based regimens, patients treated with IVT before EVT, and studies limited to near-complete or complete reperfusion after EVT, such as eTICI 2c–3. These findings suggest that the effect of adjunctive IAT is context-dependent, varying according to reperfusion quality, vascular territory, thrombolytic regimen, and patient selection.

### 3.4. Medium Vessel Thrombectomy

While EVT has been conclusively shown to improve outcomes in patients with LVO, evidence for its use in medium vessel occlusion (MeVO) remains limited. MeVO typically involves occlusions of the nondominant or codominant M2 segment, distal M3 or M4 branches of MCA, or comparable segments of the anterior or posterior cerebral artery (ACA or PCA) [60]. Such occlusions may respond poorly to IVT and, if unrecanalized, can result in substantial neurological disability and increased mortality. Nevertheless, extrapolation of the LVO treatment paradigm to MeVO warrants caution.

Two large randomized controlled trials published in 2025—the ESCAPE-MeVO and DISTAL trials—provided critical insights into the efficacy of EVT in this population. The ESCAPE-MeVO trial found that EVT did not result in significantly better 90-day functional outcomes compared with usual medical care (mRS 0–1: 41.6% vs. 43.1%, adjusted RR 0.95, 95% CI 0.79–1.15, *p* = 0.61) [60]. Similarly, the DISTAL trial reported neutral results, with no significant functional benefit associated with EVT (odds ratio 0.90, 95% CI 0.67–1.22, *p* = 0.50) [61]. The 2026 Guideline for the Early Management of Patients with Acute Ischemic Stroke also states that, for occlusions involving the nondominant proximal M2 segment of MCA, distal MCA, ACA, and PCA, EVT is classified as a Class 3 recommendation (no benefit/harm) and is not recommended for improving functional outcomes [5]. The recurrent neutral results observed across these studies are likely multifactorial. Medium vessels are more challenging to detect, potentially delaying treatment and reducing the volume of salvageable tissue. In addition, EVT for medium or distal vessels involves navigating through finer and more tortuous vasculature, where traditional thrombectomy devices may struggle to reach safely and operate effectively.

However, some investigators have argued that this may partly reflect the inclusion of a large proportion of patients with mild stroke, in whom a ceiling effect may have limited the net benefit of EVT. At the International Stroke Conference (ISC) 2026, the ORIENTAL-MeVO trial showed that EVT improved 90-day outcomes in patients with medium or distal vessel occlusion within 24 h who had moderate-to-severe deficits (58.6% vs. 46.6%, adjusted relative rate 1.24, 95% CI 1.07–1.44, *p* = 0.004), without increasing sICH or 90-day mortality. The benefit appeared greater in those with NIHSS > 8. These findings support a more selective EVT strategy in MeVO based on both occlusion site and symptom severity.

## 4. Cytoprotective Therapy

In the modern reperfusion era, cytoprotective therapy has increasingly been regarded as an important strategy to complement reperfusion therapy and promote tissue salvage. Although cytoprotection has long been a major focus in stroke research, its clinical translation has remained limited, with most studies yielding neutral or negative results. This may be partly explained by the mismatch between preclinical animal models and clinical stroke populations, delayed treatment initiation, and the fact that many early studies targeted only a single pathophysiological mechanism [62]. In recent years, however, with a growing understanding of the mechanisms underlying ischemic and reperfusion injury, cytoprotective agents such as nerinetide, edaravone dexborneol and other agents, which act on multiple molecular and cellular pathways, have re-emerged as promising therapeutic candidates. In particular, when combined with reperfusion therapy, these agents may further improve neurological outcomes.

Nerinetide is a peptide-based neuroprotective agent that acts by disrupting the interaction between postsynaptic density protein 95 (PSD-95), the GluN2B subunit of the NMDA receptor, and neuronal nitric oxide synthase [63]. In the ESCAPE-NA1 trial (*n* = 1105), nerinetide did not improve functional outcomes in the overall EVT/IVT-treated population (61.4% vs. 59.2%, adjusted RR 1.04, 95% CI 0.96–1.14, *p* = 0.35), although prespecified subgroup analyses suggested potential benefit in patients who did not receive alteplase, leading to the hypothesis that alteplase may degrade such peptide drugs [64]. To test this hypothesis, the ESCAPE-NEXT trial (*n* = 850) enrolled patients undergoing EVT without prior IVT and also reported neutral results (mRS 0–2: 45% vs. 46%, odds ratio 0.97, 95% CI 0.72–1.30, *p* = 0.82) [65]. The FRONTIER study (*n* = 532) explored the feasibility of prehospital administration within 3 h of symptom onset. Although no overall benefit was observed, adjusted analyses suggested a potential therapeutic effect in patients treated early and selected for reperfusion therapy [66]. A meta-analysis (*n* = 2487) pooling individual patient data from these three trials supported an early time-window effect, showing clinically meaningful benefits in mRS scores, stroke worsening, and infarct volume [67]. Accordingly, nerinetide may be most relevant for patients treated within 3 h of onset who are selected for reperfusion therapy.

Edaravone Dexborneol is a compound neuroprotective agent combining the free radical scavenger edaravone with the anti-inflammatory agent dexborneol [68]. In the phase 3 TASTE trial (*n* = 1165), edaravone dexborneol was associated with improved 90-day functional outcomes compared with edaravone alone (mRS 0–1: 67.18% vs. 58.97%, odds ratio 1.42, 95% CI 1.12–1.81, *p* = 0.004) [69]. These findings were reinforced by the 2024 TASTE-SL study (*n* = 914), which demonstrated similar benefits with a sublingual formulation (64.4% vs. 54.7%, odds ratio 1.50, 95% CI 1.15–1.95, *p* = 0.003) [70]. The phase 2 INSIST-ED trial (*n* = 200) showed a numerically favorable trend, although the difference was not statistically significant [71]. Then the phase 3 TASTE-2 trial confirmed that initiating edaravone dexborneol before EVT in patients within 24 h of symptom onset improved the 90-day functional independence rate compared with placebo (55% vs. 49.6%, RR 1.11, 95% CI 1.00–1.23), with a favorable safety profile [72]. Together, these studies indicate that edaravone dexborneol may enhance outcomes, with the most promising signal observed when it is initiated before EVT as part of an integrated reperfusion strategy.

Butylphthalide (DL-3-n-butylphthalide, NBP) is a pleiotropic cytoprotective agent with anti-inflammatory, antioxidative, and microcirculatory effects [73]. In the BAST trial (*n* = 1216), adjunctive NBP combined with reperfusion therapy (IVT and/or EVT) significantly increased the rate of patients achieving a favorable functional outcome at 90 days compared with placebo within 6 h of onset (56.7% vs. 44.0%, odds ratio 1.70, 95% CI 1.35–2.14, *p* < 0.001) [74].

Minocycline is a widely used, well-tolerated antibiotic that may exert neuroprotective effects by targeting post-ischemic neuroinflammation through multiple mechanisms. The recently reported EMPHASIS trial showed that oral minocycline administered within 72 h of stroke onset (a loading dose of 200 mg followed by 100 mg every 12 h for 4 days) significantly improved 90-day functional outcomes (mRS 0–1) in patients with AIS (52.6% vs. 47.4%, adjusted RR 1.11, 95% CI 1.03–1.20, *p* = 0.0061), without raising major safety concerns [75]. Although these findings represent an important step forward for anti-inflammatory strategies in stroke, their real-world applicability still requires further evaluation, particularly with respect to appropriate antibiotic stewardship and patient selection.

The STAIR XIII conference emphasized closer integration of cytoprotective strategies with reperfusion therapy [76]. Although reperfusion therapy restores blood flow, cytoprotective approaches may further improve outcomes by preserving penumbral tissue, attenuating reperfusion-related injury, and reducing secondary neuroinflammation. Their therapeutic effect may therefore depend on the interaction with reperfusion status, treatment timing, and patient selection. In this context, enhancing thrombolytic efficacy, mitigating reperfusion injury, and preventing the no-reflow phenomenon have emerged as key priorities for future research. These directions support the evolving role of cytoprotection as a complementary component of comprehensive stroke management.

## 5. Conclusions

In summary, the future of AIS treatment may lie not merely in extending therapeutic time windows or achieving recanalization in a greater number of vessels, but in redefining treatment success as effective tissue salvage rather than vascular reopening alone. The inconsistency observed across several pivotal trials suggests that, although recanalization is a prerequisite, it is not always sufficient to translate into optimal functional recovery. Future progress may therefore depend less on simply widening treatment windows or adding more therapeutic modalities and more on identifying patients with potentially reversible tissue injury, optimizing reperfusion quality, and selecting those in whom cytoprotective strategies can most effectively complement reperfusion to preserve vulnerable tissue and improve functional outcomes.

## Figures and Tables

**Table 1:** Randomized controlled clinical trials comparing intravenous thrombolysis with tenecteplase to alteplase (0.9 mg/kg) in patients with acute ischemic stroke.

	Trial Design	Participants	Intervention (Tenecteplase)	Control (Alteplase)	Participants Randomly Assigned, *n*	Primary Outcome	Main Findings
AcT (NCT03889249)	PROBE, registry-linked	Canadian AIS patients within 4.5 h, eligible for thrombolysis	0.25 mg/kg	0.9 mg/kg (10% bolus + 60 min infusion)	1600	mRS score 0–1 at 90–120 days (Non-inferiority)	Tenecteplase (0.25 mg/kg): non-inferior in terms of efficacy, similar safety profile
TRACE-2 (NCT04797013)	PROBE	Chinese AIS patients within 4.5 h, ineligible for thrombectomy	0.25 mg/kg	0.9 mg/kg (10% bolus + 60 min infusion)	1430	mRS score 0–1 at 90 days (Non-inferiority)	Tenecteplase (0.25 mg/kg) was non-inferior to alteplase for efficacy, with a similar safety profile.
ATTEST-2 (NCT02814409)	PROBE	UK patients with AIS within 4.5 h, eligible for thrombolysis	0.25 mg/kg	0.9 mg/kg (10% bolus + 60 min infusion)	1858	90-day mRS distribution (Non-inferiority)	Tenecteplase (0.25 mg/kg) did not differ significantly from alteplase in efficacy or safety.
TASTE (ACTRN12613000243718)	PROBE	International AIS patients within 4.5 h with perfusion imaging target mismatch	0.25 mg/kg	0.9 mg/kg (10% bolus + 60 min infusion)	680	mRS score 0–1 at 90 days (Non-inferiority)	Tenecteplase (0.25 mg/kg) was as effective as alteplase and demonstrated a comparable safety profile.
ORIGINAL (NCT04915729)	PROBE	Chinese AIS patients within 4.5 h, NIHSS 1–25, eligible for thrombolysis	0.25 mg/kg	0.9 mg/kg (10% bolus + 60 min infusion)	1489	mRS score 0–1 at 90 days (Non-inferiority)	Tenecteplase (0.25 mg/kg): equally safe and non-inferior in terms of efficacy

PROBE: Prospective, Randomized, Open-label, Blinded Endpoint. mRS: modified Rankin scale. NIHSS: National Institutes of Health Stroke Scale.

**Table 2:** Characteristics of randomized controlled trials evaluating EVT in extended time windows and large-core infarction.

EVT in Extended Time Window						
	Trial Design	Population	Intervention	Control	Primary Outcome	Main Findings
DAWN (NCT02142283)	RCT, open-label, multicenter, phase 3	- Anterior circulation LVO - Within 6–24 h from last known well - Clinical–imaging mismatch between deficit severity and infarct volume	EVT + medical therapy	Medical therapy alone	90-day mRS score 0–2	EVT was associated with better 90-day functional outcomes in selected patients treated 6–24 h after last known well.
DEFUSE-3 (NCT02586415)	RCT, open-label, multicenter, blinded endpoint, phase 3	- Anterior circulation LVO - Within 6–16 h from last known well - Perfusion imaging showing salvageable tissue	EVT + medical therapy	Medical therapy alone	90-day ordinal mRS score	EVT plus standard medical therapy improved 90-day functional outcomes in patients selected by perfusion imaging within 6–16 h.
ATTENTION (NCT04751708)	RCT, multicenter, phase 3	- Posterior circulation LVO - Within 12 h of estimated occlusion time - Moderate-to-severe stroke	EVT + medical therapy	Medical therapy alone	90-day mRS score 0–3	EVT resulted in a higher likelihood of favorable functional outcome in BAO, despite increased hemorrhagic and procedural risks.
BAOCHE (NCT02737189)	RCT, open-label, multicenter, phase 3	- Posterior circulation LVO - Within 6–24 h of symptom onset - No large baseline posterior-circulation infarct	EVT + medical therapy	Medical therapy alone	90-day mRS score 0–3	EVT improved 90-day functional outcomes in patients with BAO treated 6–24 h after symptom onset.
EVT in Large-Core Infarction						
	Trial Design	Population	Intervention	Control	Primary Outcome	Main findings
RESCUE-Japan LIMIT (NCT03702413)	RCT, open-label, blinded endpoint, multicenter, phase 3	- Anterior circulation LVO - Large infarct (ASPECTS 3–5) - Within 6 h of onset (or ≤24 h with FLAIR negative)	EVT + medical therapy	Medical therapy alone	90-day mRS score 0–3	The EVT group had a significantly higher proportion of mRS 0–3 at 90 days with increased risk of intracranial hemorrhage.
TENSION (NCT03094715)	RCT, open-label, blinded endpoint, multicenter, phase 3	- Acute anterior circulation LVO - Large infarct (ASPECTS 3–5) - Within 12 h of onset	EVT + medical therapy	Medical therapy alone	90-day mRS distribution	EVT improved 90-day mRS distribution; the benefit was sustained at 12 months with improved survival and quality of life.
LASTE (NCT03811769)	RCT, open-label, blinded endpoint, phase 3	- Anterior circulation LVO - Large infarct on MRI or CT (ASPECTS 0–5) - Within 6.5 h of onset (or MRI FLAIR negative)	EVT + medical therapy	Medical therapy alone	90-day mRS distribution	EVT improved 90-day mRS distribution and reduced mortality; the benefit was sustained at 180 days with improved quality of life.
ANGEL-ASPECT (NCT04551664)	RCT, open-label, blinded endpoint, multicenter, phase 3	- Anterior circulation LVO - Large infarct (ASPECTS 3–5 or core 70–100 mL) - Within 24 h of onset	EVT + medical therapy	Medical therapy alone	90-day mRS distribution	EVT improved 90-day mRS distribution and functional independence but was associated with a higher rate of intracranial hemorrhage.
SELECT2 (NCT03876457)	RCT, open-label, blinded endpoint, multicenter, phase 3	- Anterior circulation LVO - Large infarct (ASPECTS 3–5 or core ≥50 mL) - Within 24 h of onset	EVT + medical therapy	Medical therapy alone	90-day mRS distribution	EVT improved 90-day mRS distribution and increased functional independence rate. sICH rate was low and similar between groups.
TESLA (NCT03805308)	Bayesian adaptive RCT, open-label, blinded endpoint, phase 3	- Anterior circulation LVO - Large infarct on NCCT (ASPECTS 2–5) - Within 24 h of onset	EVT + medical therapy	Medical therapy alone	90-day UW-mRS mean score	EVT did not significantly reduce the 90-day UW-mRS mean score.

RCT: Randomized Controlled Trial. LVO: Large Vessel Occlusion. BAO: Basilar Artery Occlusion. ASPECTS: Alberta Stroke Program Early Computed Tomography Score. NCCT: Noncontrast Computed Tomography. mRS: modified Rankin scale.UW-mRS: Utility-Weighted modified Rankin scale.

**Table 3:** Characteristics of randomized controlled trials evaluating bridging, adjunct intra-arterial thrombolysis after EVT, and endovascular therapy for medium-vessel occlusion in acute ischemic stroke.

Bridging Therapy and Adjunctive Intra-Arterial Thrombolysis						
	Trial Design	Population	Intervention	Control	Primary Outcome	Main Findings
BRIDGE-TNK (NCT04733742)	RCT, multicenter, phase 3	- AIS due to LVO (ICA, M1/M2, or vertebrobasilar occlusion) - Within 4.5 h of onset	IV tenecteplase before EVT	EVT alone	90-day mRS score 0–2	IV tenecteplase before EVT improved 90-day functional independence compared with EVT alone.
CHOICE (NCT03876119)	RCT, double-blinded, phase 2b	- AIS due to LVO (ACA, MCA, or PCA occlusion) - Successful reperfusion after EVT (eTICI ≥ 2b50) - Within 24 h of onset	IA alteplase after successful EVT	Placebo after successful EVT	90-day mRS score 0–1	IA alteplase after successful EVT improved excellent functional outcome at 90 days.
POST-UK (ChiCTR2200065617)	RCT, open-label, blinded endpoint, multicenter, phase 3	- AIS due to LVO (intracranial ICA, M1, or M2 occlusion) - Near-complete to complete re- perfusion after EVT (eTICI 2c-3) - Within 24 h of onset	IA urokinase after EVT	EVT alone	90-day mRS score 0–1	IA urokinase after successful EVT did not significantly improve disability-free survival at 90 days
POST-TNK (ChiCTR2200064809)	RCT, open-label, multicenter, phase 3	-AIS due to LVO (intracranial ICA, M1, or M2 occlusion) - Near-complete to complete reperfusion after EVT (eTICI 2c-3) - Within 24 h of onset	IA tenecteplase after EVT	EVT alone	90-day mRS score 0–1	IA tenecteplase after successful EVT did not significantly improve excellent functional outcome at 90 days.
ANGEL-TNK (NCT05624190)	RCT, open-label, blinded endpoint, multicenter, phase 3	- Acute anterior-circulation LVO - Successful reperfusion after EVT (eTICI 2b-3) - Within 4.5–24 h from last known well -No prior IVT	IA tenecteplase after successful EVT	EVT alone	90-day mRS score 0–1	IA tenecteplase after successful EVT increased the likelihood of excellent neurological outcome at 90 days without increasing sICH or mortality.
PEARL (NCT05856851)	RCT, open-label, blinded endpoint, multicenter, phase 3	- AIS due to anterior-circulation LVO (intracranial ICA, M1, or M2 occlusion) - Successful reperfusion after EVT (eTICI ≥ 2b50) -Within 24 h of onset	IA alteplase after successful EVT	Standard treatment after EVT	90-day mRS score 0–1	IA alteplase after successful EVT reperfusion improved excellent functional outcome at 90 days.
ATTENTION-IA (NCT05684172)	RCT, multicenter	- Posterior circulation LVO (vertebral artery V4, basilar artery, or P1 segment of PCA occlusion) - Successful reperfusion after EVT (eTICI 2b50-3) - Within 24 h of onset	IA tenecteplase after successful EVT	EVT alone	90-day mRS score 0–1	IA tenecteplase after successful posterior-circulation EVT did not significantly improve excellent functional outcome at 90 days.
EVT for Medium-Vessel Occlusion						
	Trial Design	Population	Intervention	Control	Primary Outcome	Main findings
ESCAPE-MeVO (NCT05151172)	RCT, open-label, multicenter, phase 3	- Medium-vessel occlusion - Within 12 h from last known well - Favorable baseline noninvasive brain imaging	EVT + best medical care	Best medical care alone	90-day mRS score 0–1	EVT did not improve 90-day functional outcomes in patients with medium-vessel occlusion.
DISTAL (NCT05029414)	RCT, assessor-blinded, multicenter	- Medium or distal vessel occlusion - Within 24 h from last known well - Dominant M2 occlusion excluded	EVT + best medical care	Best medical care alone	90-day mRS distribution	EVT did not reduce disability or death at 90 days in patients with medium or distal vessel occlusion.

RCT: Randomized Controlled Trial. LVO: large-vessel occlusion. ICA: Internal Carotid Artery. ACA: Anterior Cerebral Artery. MCA: Middle Cerebral Artery. PCA: Posterior Cerebral Artery. eTICI: expanded Thrombolysis in Cerebral Infarction. mRS: modified Rankin Scale.

## Data Availability

No new data were created or analyzed in this study. Data sharing is not applicable to this article.

## Abbreviations

ACA	Anterior Cerebral Artery
AIS	Acute Ischemic Stroke
ASPECTS	Alberta Stroke Program Early Computed Tomography Score
BAO	Basilar artery occlusion
CDE	Center for Drug Evaluation
eTICI	expanded Thrombolysis in Cerebral Infarction
EVT	Endovascular Thrombectomy
IAT	Intra-arterial Thrombolysis
ICA	Internal Carotid Artery
IVT	Intravenous Thrombolysis
LVO	Large Vessel Occlusion
NBP	DL-3-n-butylphthalide
MCA	Middle Cerebral Artery
MeVO	Medium Vessel Occlusion
mRS	modified Rankin scale
NIHSS	National Institute of Health Stroke Scale
NCCT	Noncontrast Computed Tomography
PCA	Posterior Cerebral Artery
PROBE	Prospective, Randomized, Open-label, Blinded Endpoint
RD	Risk Difference
rhPro-UK	Recombinant Human Prourokinase
RR	Risk Ratio
sICH	Symptomatic Intracranial Hemorrhage
UW-mRS	Utility-weighted modified Rankin scale
